# Microbiota Composition May Predict Anti-Tnf Alpha Response in Spondyloarthritis Patients: an Exploratory Study

**DOI:** 10.1038/s41598-018-23571-4

**Published:** 2018-04-03

**Authors:** Thomas Bazin, Katarzyna B. Hooks, Thomas Barnetche, Marie-Elise Truchetet, Raphaël Enaud, Christophe Richez, Maxime Dougados, Christophe Hubert, Aurélien Barré, Macha Nikolski, Thierry Schaeverbeke

**Affiliations:** 1 Univ. Bordeaux, INRA, Mycoplasmal and chlamydial infections in humans, EA 3671, 33000 Bordeaux, France; 2 Bordeaux Hospital University Center, Department of Hepato-gastroenterology, 33600 Pessac, France; 3 Univ. Bordeaux, Bordeaux Bioinformatics Center, 33000 Bordeaux, France; 4 Univ. Bordeaux, CNRS, Immunoconcept, UMR 5164, 33000 Bordeaux, France; 5 Bordeaux Hospital University Center, Department of Rheumatology, 33000 Bordeaux, France; 6 Univ. Bordeaux, Centre de Recherche Cardio-Thoracique de Bordeaux, U1045, FHU ACRONIM, Laboratoire 8 de Parasitologie-Mycologie, F-33000 Bordeaux, France; 7 CHU Bordeaux, Unité d’Hépatologie, Gastroentérologie et Nutrition Pédiatriques, CRCM Pédiatrique, Service 10 de Rhumatologie, Service d’Exploration Fonctionnelle Respiratoire, 33000 Bordeaux, France; 8 INSERM, Centre de Recherche Cardio-Thoracique de Bordeaux, U1045, F-33000 Bordeaux, France; 9 AP-HP, Cochin Hospital University Center, Department of Rheumatology, 75014 Paris, France; 10 Univ. Bordeaux, INSERM, Rare Diseases, genetic and metabolism, U1211, 33000 Bordeaux, France; 11 Univ. Bordeaux, Genome Transcriptome Facility of Bordeaux, 33000 Bordeaux, France; 12 Univ. Bordeaux, CNRS, LaBRI, UMR 5800, 33400 Talence, France

## Abstract

Spondyloarthritis (SpA) pathophysiology remains largely unknown. While the association with genetic factors has been established for decades, the influence of gut microbiota is only an emerging direction of research. Despite the remarkable efficacy of anti-TNF-α treatments, non-responders are frequent and no predictive factors of patient outcome have been identified. Our objective was to investigate the modifications of intestinal microbiota composition in patients suffering from SpA three months after an anti-TNF-α treatment. We performed 16S rDNA sequencing of 38 stool samples from 19 spondyloarthritis patients before and three months after anti-TNF-α treatment onset. SpA activity was assessed at each time using ASDAS and BASDAI scores. Some modifications of the microbiota composition were observed after three months of anti-TNF-α treatment, but no specific taxon was modified, whatever the clinical response. We identified a particular taxonomic node before anti-TNF-α treatment that can predict the clinical response as a biomarker, with a higher proportion of Burkholderiales order in future responder patients. This study suggests a cross-influence between anti-TNF-α treatment and intestinal microbiota. If its results are confirmed on larger groups of patients, it may pave the way to the development of predictive tests suitable for clinical practices.

## Introduction

Spondyloarthritis (SpA) pathophysiology involves a genetic background, characterized by a strong association with the HLA-B27 genotype and a weak link with up to 40 other genes (IL-23R, ERAP1, TNFRSF15…)^1^. The role of some environmental factors has also been shown, such as smoking^2^. More recently, a large body of evidence has emphasized the implication of the gut in the SpA pathophysiology, and more specifically of an intestinal dysbiosis. In HLA-B27 transgenic rats, the germ-free animals failed to develop the disease phenotype that was restored by the introduction of bacteria in food supply, specifically with some bacterial cocktails containing *Bacteroidetes* species^3–5^. In human, overlap between SpA and inflammatory bowel disease (IBD) is common: about 5–10% of SpA patients develop IBD, while up to 30% of IBD patients may develop inflammatory arthritis^6,7^; moreover up to 60% of patients with SpA present microscopic gut inflammation^8,9^. In addition, clinical remission of SpA is always associated with normal digestive histology, while the persistence of rheumatic symptoms is mostly associated with a persistent intestinal inflammation^10^. These data suggest a close physiopathological link between gut inflammation and SpA, the recent demonstration of the crucial role of the gut microbiota in IBD raising the same query in SpA^11^.

Five Next-Generation DNA Sequencing (NGS) studies compared the gut microbiota of patients suffering from SpA spectrum disease with that of healthy controls. Stoll *et al*. examined gut microbiota of 25 pediatric patients with enthesitis-related arthritis and 13 healthy controls^12^. They showed that these patients exhibited a significant reduction of *Faecalibacterium prausnitzii*, as observed in Crohn’s disease. Costello *et al*. studied terminal ileal biopsies obtained during colonoscopy in nine ankylosing spondylitis (AS) patients and nine controls. They observed an increase in *Lachnospiraceae*, *Veillonellaceae*, *Prophyromonadaceae* and *Bacteroidaceae* and a decrease in *Ruminococcaceae* and *Rikenellaceae* in patients compared with healthy control subjects^13^. Still in AS, Wen *et al*. performed a quantitative metagenomic study on 97 AS patients and 114 healthy controls. They found in AS patients an increase in the abundance of *Prevotella melaninogenica*, *Prevotella copri*, and *Prevotella* sp. C561 and *Bifidobacterium* genus, and a decrease in *Bacteroides* spp.^14^.

Concerning SpA, Tito *et al*. performed investigation of bacterial composition from ileal and colonic biopsies from 27 SpA patients^15^. They found different microbial profiles associated with the status of inflammation in the tissue and observed positive correlation between abundance of *Dialister* sp. and Ankylosing Spondylitis Disease Activity Score (ASDAS). More recently, Breban *et al*. compared microbiota composition of 87 SpA patients to that of 69 controls. Microbial diversity was lower in SpA patients, with a marked increase of *Ruminococcus gnavus*^16^.

Tumour necrosis factor alpha (TNF-α) inhibitors have revolutionized the treatment of SpA patients who failed to respond to NSAID and conventional Disease-Modifying Anti-rheumatic Drugs (DMARDs)^17,18^. However, non-response to this biotherapy is a major concern for clinicians, as only about 30 to 50% of patients exhibit a meaningful clinical response^19,20^. The mechanism of action of these widely-used molecules is still a matter of debate. The most commonly accepted hypothesis is that, by acting on host immune cells, they down-regulate the inflammatory cascade leading to clinical symptoms^21^.

In this context, the aim of our study was to investigate the modification of the intestinal microbiota three months after the introduction of an anti-TNF-α treatment and to look for a relationship between the characteristics of the microbiota composition at M0 and the clinical response to treatment.

## Methods

All methods were performed in accordance with the relevant guidelines and regulations.

### Study design and patients

We conducted a bicentric prospective observational hospital-based exploratory study from September 2013 through August 2015 at the Rheumatology departments of the Bordeaux University Hospital and of Cochin University Hospital in Paris. Patients’ inclusion criteria were as follows: (i) at least 18 years of age, with a diagnosis of axial only or axial and peripheral SpA fulfilling the ASAS imaging criteria, (ii) naïve to anti-TNF-α, justifying the initiation of an anti-TNF-α treatment according to current guidelines (recommendations of the French Society for Rheumatology)^22^ and (iii) affiliated to health insurance. Exclusion criteria were as follows: patients with (i) an inflammatory bowel disease, (ii) an history of bowel resection or digestive stoma, or under antibiotic treatment in the three months before inclusion, (iii) any contra-indication to anti-TNF-α therapy, (iv) refusal to sign the informed consent or linguistic or cognitive difficulties that did not allow a full understanding of the consent form, (v) pregnancy or breastfeeding, or the refusal to follow an effective contraception method for all the study duration (for women).

Informed consent was obtained from study participants. The non-interventional character of this study has been approved by the ethical committee “CPP Sud-Ouest et Outremer III”. All enrolled patients were followed in the service of rheumatology at Bordeaux University Hospital or Cochin University Hospital, where a first anti-TNF-α treatment for SpA was initiated. Clinical characteristics of SpA were registered at two times of stool sampling, at enrollment (M0) and after three months of biotherapy (M3); activity was assessed using ASDAS and BASDAI scores^23^. The lack of clinical response was defined by an ASDAS improvement ≤1, partial response by an ASDAS improvement between 1 and 2 and clinical response by an ASDAS improvement ≥2. CRP levels and HLA-B27 status were obtained by systematic blood tests.

The choice of anti-TNF-α drug and dosage was left to the discretion of the clinicians in accordance with standard practices. Possible combination with other treatments (immunosuppressant agents, corticosteroids, non steroidal anti-inflammatory drugs) has been left up to the clinicians and recorded in the Supplementary Table 1.

### Sample collection and DNA extraction

Stool samples were collected at M0 and M3 and frozen at −80 °C within 24 hours after collection. DNA contained in feces was extracted with Dneasy® Blood & Tissue Kit (Qiagen) according to DNeasy Blood & tissue handbook (Qiagen). In order to optimize the extraction for Gram-positive bacteria, we added a combination of lysostaphin and lysozyme (20 mg/ml in lysis solution). We then used silica columns, provided with the previously described kit, to separate DNA from other prokaryotic cells components.

### 16S amplification

16S DNA from 38 samples (19 × 2) was amplified using 2x Phusion GC Master mix and primers 515 F and 806 R (5′CTTTCCCTACACGACGCTCTTCCGATCTGTGCCAGCMGCCGCGGTAA and 5′GGAGTTCAGACGTGTGCTCTTCCGATCTGGACTACHVGGGTWTCTAAT, respectively), targeting variable V4 region of 16S bacterial DNA. Maximal expected amplicon length of 347 bp was compatible with direct paired-end MiSeq Illumina sequencing. The DNA was amplified using Master Mix Phusion GC Buffer (New England Biolabs^®^). PCR conditions were as follows: 30 ng of DNA, two primers with final concentration 10 µM each, 25 µl of Master Mix Phusion GC Buffer, and completion with water leading to a final volume of 50 µl. Cycle conditions were as follows: 1 cycle of 98 °C, 30 s (hot start activation); 25 cycles of 98 °C,10 s (denaturation)/60 °C, 30 s (hybridation)/72 °C, 45 s (elongation); and 72 °C during 7 min (final elongation). Then, purification with magnetic beads was performed (Beckman Agencourt^®^ AMPure).

### Library build and sequencing

Resulting libraries were pooled, normalized and denatured according to Illumina protocol. Samples were then deposited on a MiSeq flowcell 15 M and sequenced using the MiSeq Illumina^®^ sequencer at the Genome Transcriptome facility of the University of Bordeaux, generating paired-end reads of 2 × 250 bp. Raw data have been deposited in the ENA sequence read archive (ENA accession number PRJEB23107).

### 16S bioinformatic sequence analysis

Raw reads were quality filtered using the NGS QC toolkit (version 2.3.3)^24^ and kept if both of the paired reads passed the filter criteria. The cutoff for quality score was >20 Q30 and >70% of the total read length should have high-quality bases. Possible human contamination was filtered out by mapping the remaining high quality reads against the human genome (*Homo sapiens* alternate assembly HuRef GCF_000002125.1) using BWA (version 0.7.12)^25^ with default parameters. Possible chimeric sequences were further identified and eliminated using DECIPHER (version 2.14.1)^26^. Remaining reads were aligned with BWA against the 16S sequences contained in the GreenGenes database (v13.5)^27^ keeping all the hits (Supplemental Table 2).

Two complementary methods for analyzing microbiota composition were used: operational taxonomic units (OTU) and taxonomic assignment with tango. First remaining reads after filtering were classified using USEARCH_global method of VSEARCH v2.3.0^28^ against 16S OTUs from GreenGenes database with a 97% identity threshold. We have further analysed microbiota richness of our samples by building rarefaction curves based on the OTU computation with the R package vegan^29^. Microbiota alpha diversity as expressed by Shannon and Simpson indexes was computed from the OTU occurrence matrix. To calculate phylogenetic beta diversity, we used a weighted UniFrac metric^30^ implemented by R package phyloseq.^31^ by inputting OTU table (Supplemental Table 3) and GreenGenes OTU tree. Then, to robustly deal with the reads mapped to multiple taxa, we used Tango for taxonomic assignment with *q*-value parameter set to 0.5^32^. This approach allows using ambiguous reads by assigning them to higher taxonomic ranks. Consequently, the number of reads assigned to the root was equal to the total number of high quality reads of the sample. Number of reads assigned at every taxonomic node was normalized by the total number of reads in each sample. Normalized counts of all samples were combined in a global occurrence matrix (Supplemental Table 4).

### Prediction of bacterial function

Biological function of the assigned microbiota was predicted using predictive functional metagenome PICRUSt method^33^. Briefly, OTU table was normalized by copy number using precomputed tables of gene counts from GreenGenes. The mean of weighted nearest sequenced taxon index scores of 0.082 ± 0.018 suggest a good imputation quality. KEGG orthologs prediction was used to identify gene families. In total 328 KEGG pathways were imputed. Pathways with no proportion higher than 90% were removed, leaving 279 pathways. Pathways were analyzed using DESeq. 2, a *p*-value threshold of 5% and ratio change of more than 5% was considered significant.

### Statistical analysis

Nonparametric Wilcoxon-Mann-Whitney test was used to compare quantitative variables between groups. Correlations were calculated using Spearman method. Correction for multiple-testing was performed using Benjamini Hochberg test. LEfSe method was used to discover metagenomic biomarkers^34^.

### Ethics approval and consent to participate

Ethical committee CPP Sud-Ouest et Outremer III; informed consent was sought from study participants.

### Availability of data and material

The datasets supporting the conclusions of this article (raw data) are available in the ENA sequence read archive (ENA accession number PRJEB23107).

## Results

### Patients

Nineteen patients were recruited. Given the rarefaction curves, we excluded patient P6 since the asymptote was not reached in the M0 sample for this patient (Supplemental Fig. 1). Consequently, the analysis of microbiota sequencing was performed for 18 out of 19 patients who participated in the study (Table 1 and Supplemental Table 1) and P6 does not appear in figures.

**Table 1:** Demographic and clinical characteristics of patients.

Patients N	18
Females	5 (28%)
Age (years)*	37 ± 14
Disease duration (years)*	2 ± 14
HLA-B27 positive	15 (83%)
Localisation	
Axial	3 (17%)
Axial and peripheral	15 (83%)
M0	
CRP (mg/L)*	7 ± 10.7
ASDAS*	2.9 ± 0.8
BASDAI*	4.9 ± 1.7
M3	
CRP (mg/L)*	2 ± 1.2
ASDAS*	1.4 ± 0.9
BASDAI*	2.0 ± 2.3
Response at M3	
non responder (NR)	8 (44%)
partial responder (PR)	5 (28%)
responder (R)	5 (28%)
Δ ASDAS*	1.3 ± 1.2

*Medians ± SD.

None of them has been treated with any of the biologic DMARDs prior to the study and they were considered eligible for anti-TNF-α treatment. Nine patients were referred by Cochin Hospital and nine by Bordeaux Hospital; 13 men and 5 women presented with axial Ankylosing Spondylitis (N = 3) or axial and peripheral Ankylosing Spondylitis (N = 15). Fifteen of them were HLA-B27 positive. Prior to the beginning of the study, the median Ankylosing Spondylitis Disease Activity Score (ASDAS) value was 2.9 ± 0.8. Fifteen patients received etanercept, two received adalimumab and one received infliximab. At time M3, eight patients were determined not to have responded to the treatment (ASDAS improvement ≤1), whereas five patients exhibited substantial improvement (Δ ASDAS ≥1.1) and another five patients exhibited a major positive response (Δ ASDAS ≥2). BASDAI and ASDAS scores were higher in non-responding patients (Table 2, *p*-value = 0.007 and *p*-value = 0.048, respectively). CRP levels were higher in responding patients (*p*-value = 0.012). No difference was found between the groups in terms of sex ratio, age, disease duration or HLA-B27 status.

**Table 2:** Demographic and clinical characteristics of patients according to clinical response.

Clinical response	R (n = 5)	PR (n = 5)	NR (n = 8)
Females	0 (0%)	2 (40%)	3 (37%)
Age (years)*	24 ± 10.1	40 ± 19.8	37 ± 9.7
Disease duration (years)*	2 ± 1.5	7 ± 23.3	1 ± 10.6
HLA-B27 positive	4 (80%)	4 (80%)	7 (87%)
Localisation			
Axial	3 (17%)	4 (17%)	5 (17%)
Axial and peripheral	15 (83%)	16 (83%)	17 (83%)
CRP (mg/L) at M0*	12 ± 15.5	8 ± 4.8	2 ± 3.2
ASDAS at M0*	4.0 ± 0.6	2.4 ± 0.4	2.2 ± 0.8
BASDAI at M0*	1.0 ± 0.4	1.8 ± 0.6	5.0 ± 2.2
Δ ASDAS*	2.8 ± 0.5	1.3 ± 0.3	0.1 ± 0.7

*Medians ± SD.

### Microbiota composition description

**Figure 1:**
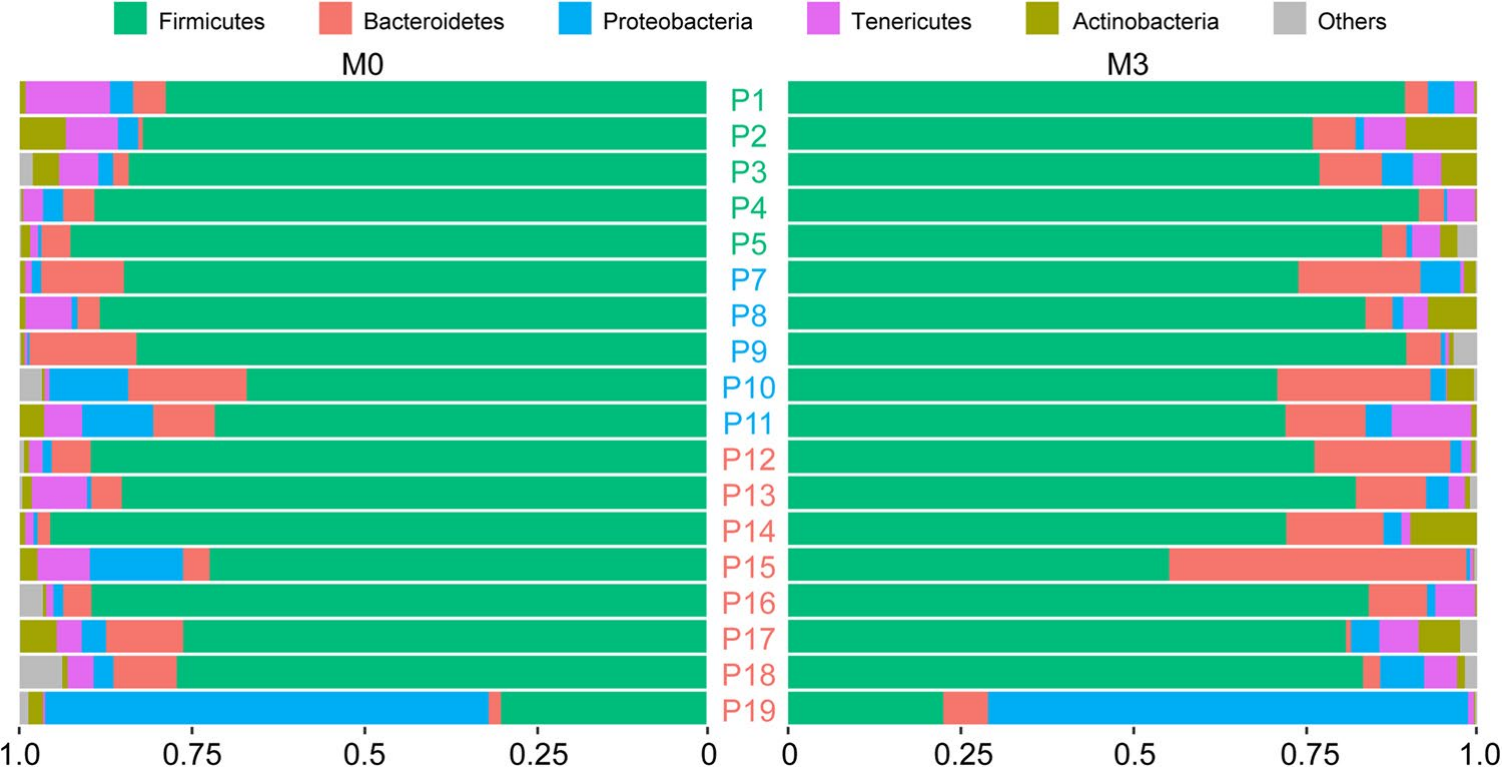
Proportion of reads assigned to different phyla. For each patient reads assigned with Tango were summed up at the phylum level. Proportions of reads belonging to five dominant phyla coloured according to the legend above are shown. Each row corresponds to one patient at M0 and M3, left and right, respectively. P1-P5 responders (green), P7-P11 partial responders (blue), P12-P19 non responders (red). Median proportions per phylum ± SD are as follows: Firmicutes − 0.82 ± 0.15, Bacteroides − 0.05 ± 0.08, Tenericutes − 0.03 ± 0.03, Proteobacteria 0.02 ± 0.15.

The median number of high-quality reads per patient was 913,431 (from 223,961 to 2,131,801). After removing singletons, reads were assigned to 24,732 unique OTUs. We used the taxonomic assignment to compare the profiles of patients’ microbiota at a phylum-level (Fig. 1). The fecal microbiota of most patients was characterized by a very high proportion of Firmicutes followed by Bacteroidetes, Tenericutes and Proteobacteria. Patient 19, who exhibited the most aggravating disease despite treatment as shown by negative ΔASDAS score and increased inflammation confirmed by a higher level erythrocyte sedimentation rate at M3 *vs* M0, (Supplementary Table 1), exhibited a clearly different microbiota profile with more Proteobacteria than other patients at M0 and M3.

### Effect of the anti- TNF-α treatment on microbiota composition

First we compared the global composition of the fecal microbiota at M0 and M3. For all patients as well as for responder or non-responder subgroups, some modifications were observed after treatment, but differences were not significant after multiple-test correction.

Subsequently, we compared the microbiota composition before and after treatment at the taxonomic levels: order, family, genus and species. There were a number of taxa that differ in proportion at the two time-points (Supplementary Table 4), with four decreasing and 15 increasing at M3. These differences were not significant after multiple-test correction. In addition, no characteristic for M0 or M3 microbiota composition was observed using LEfSe method.

**Figure 2:**
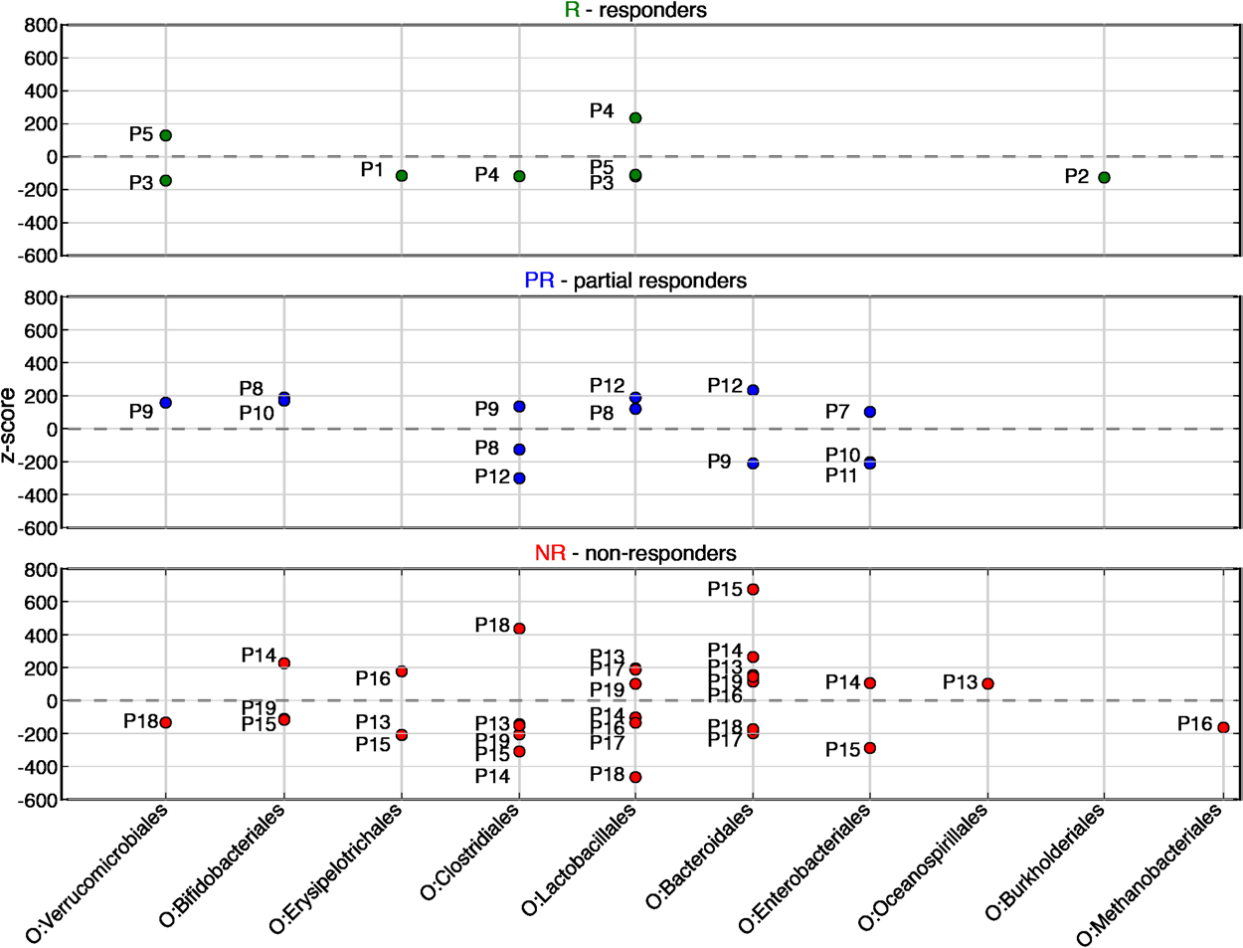
z-scores analysis at the order level. Each point represents z-score between M0 and M3 for one order for one patient. Dots above the zero black dotted represent an increase in the corresponding taxa’s proportion in the corresponding patient’s gut microbiome after the TNF alpha treatment, while dots below this line represent a decrease. Reads assigned with Tango were summed up for each patient at the order level and normalized. The *z*-scores were calculated between proportions of reads of each order at M0 and M3 (relative to the total number of reads in the sample) for each patient and filtered by $|z| > 100$. P1-P5 responders (green), P7-P11 partial responders (blue) P12-P19 non responders (red).

Still using taxonomic assignment, we then compared the variation of proportion of each taxa between M0 and M3 for each patient with the mean value of variation in all patients. This comparison is expressed as a *z*-score, which is the number of standard deviations that separate the value of interest from the mean of all the values. A value below the mean gives a negative z-score, indicating a significant decrease of the taxa when a value above the mean gives a positive z-score, corresponding to an increased proportion of the taxa. Ratio-normalized reads assigned with Tango were summed at the order level and *z*-scores were calculated between M0 and M3 for each patient and filtered by $|z| > 100$ (see Supplemental Table 5). Patients that responded well to treatment had few taxa changing after treatment, and changes were moderate, with most of taxa (6 out of 8) being reduced. Whereas for each of the non-responding patients many bacterial orders exhibited drastic changes (Fig. 2), the corresponding taxa increased or decreased chaotically in their proportion. These results suggest that patients who did not respond to anti-TNF-α treatment had high disease activity and unstable microbiota composition.

**Figure 3:**
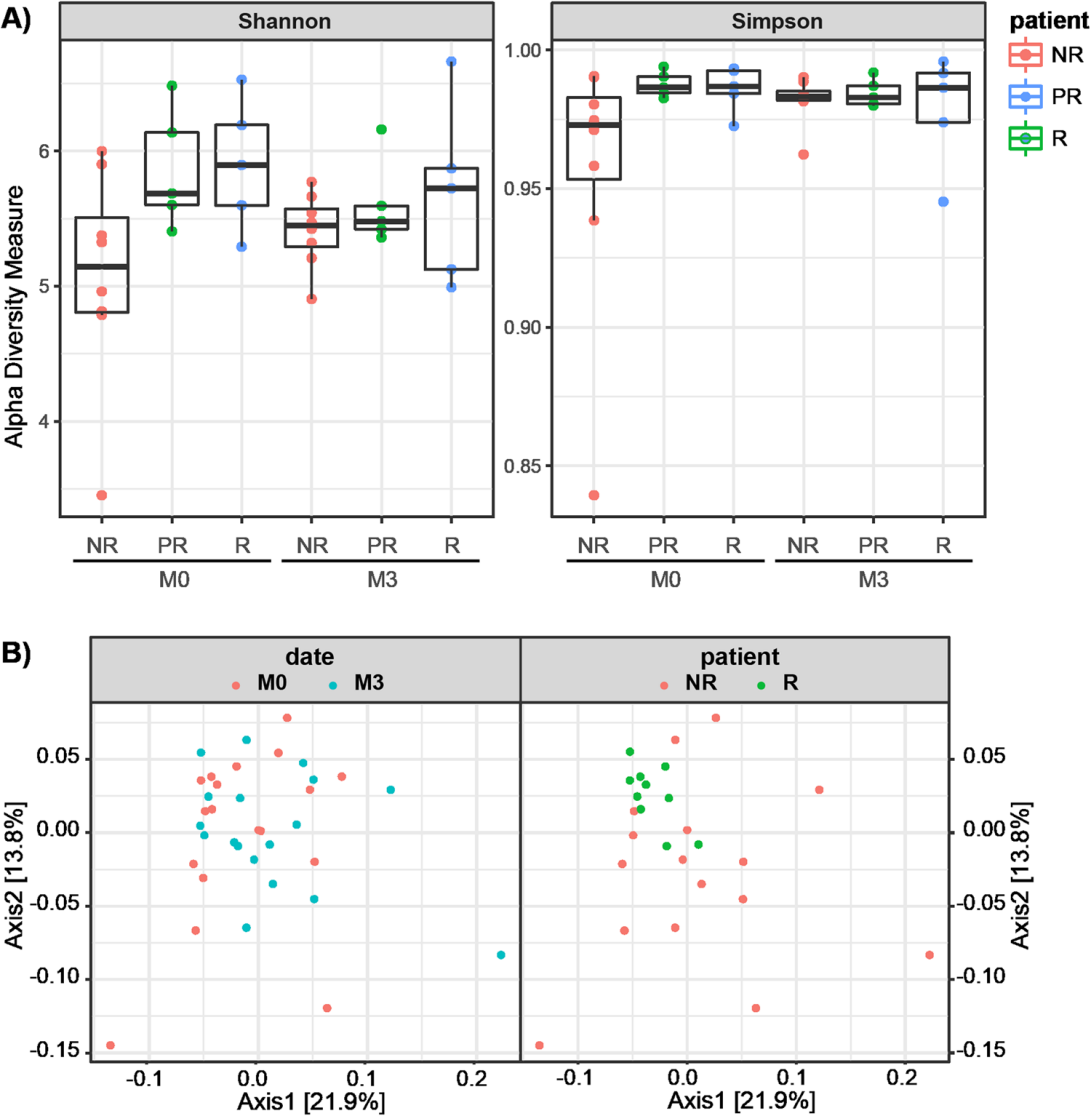
Diversity plots for microbiota patient samples. (**A**) Shannon and Simpson diversity indices calculated based on OTU analysis for each type of patient at M0 and M3. Shannon index for NR is significantly different than for R at M0 (two-tailed *t*-test with unequal variance, p-value = 0.04). (**B**) PCoA plot of β-diversity calculated by weighted UniFrac distances on OTU occurrence table. Left graph is coloured by the date of the sample. Timepoints M0 and M3 do not form separate clusters (ANOSIM, R = −0.011, p-value 0.641). Right plot is colored by the type of patient’s response (partial responders are removed for clarity). Patients with different level of response form significant clusters (ANOSIM, R = 0.1032, p-value 0.042).

Finally we analyzed the alpha and beta-diversity of the samples. Alpha diversity evaluates within-community diversity, i.e. diversity at the scale of one sample. Beta-diversity compares the composition of different communities, i.e. of different samples. Upon comparing alpha diversity as assessed by Shannon and Simpson indices we observed a difference between responder (R) and non-responder (NR) patients at M0 (Fig. 3A). The clinical response to anti-TNF-α treatment observed for a given patient at M3 correlated with the observed sample diversity at M0 (Spearman r = 0.54, *p*-value = 0.022). This suggests that patients with reduced initial microbiota diversity are more likely to fail to the anti-TNF-α treatment. However, the treatment abolishes the differences among patient groups as shown by the absence of any differences among the Shannon and Simpson indices calculated at M3, suggesting that, independently to the clinical response, anti-TNF-α treatment was able to restore the fecal microbial diversity in all patients. There was no relationship between any specific drug (etanercept, adalimumab and infliximab) or clinical feature of the disease (axial, peripheral, severity…) and microbiota diversity at the beginning or at the end of the study. When phylogenetic beta-diversity, assessed by weighted UniFrac, was used to establish microbiota distances among patients, there was no apparent grouping of patients before and after treatment (Fig. 3B, left graph). In contrast, microbiota profiles of R (M0 + M3) appeared to aggregate compared to non-responders (Fig. 3B, right graph). Microbiota within responding patients appeared to be more uniform compared to that from non-responding patients, the latter being revealed as a random scattering of the points on the PCoA plot (Fig. 3B, right graph).

### Microbial composition can predict response to anti-TNF-α treatment

We focused on the two subgroups of patients - those strongly responding to anti-TNF-α treatment (R, Δ ASDAS ≥2, n = 5) and those showing no response (NR, Δ ASDAS ≤1, n = 8) and we looked for the microbiota composition at M0 that could be predictive of the treatment outcome. We found multiple taxa at different levels to be differentially present between R and NR at M0, however after multiple test correction these results became not significant (Supplementary Table 5).

**Figure 4:**
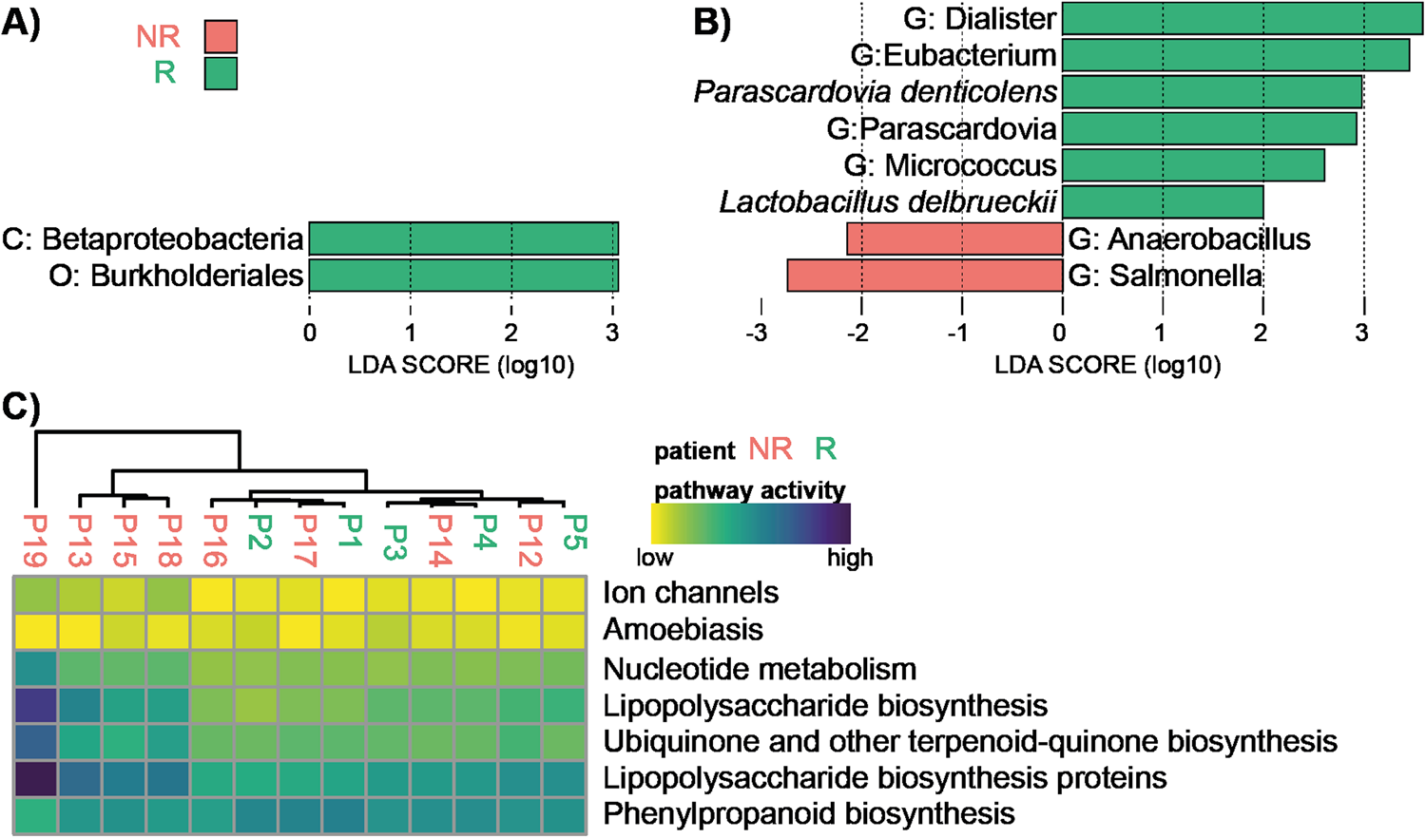
Biomarkers of responders and non-responders. LEfSE analysis distinguishing characteristics of taxonomic composition of responders and non-responders at M0 (**A**) and M3 (**B**). Biomarkers are coloured according to the legend. Taxa of higher level than species are denoted as follows: G: genus, C: class and O: order. (**C**) Heatmap showing most diversely activated pathways between responders and non-responders at M0 as predicted by PICRUSt.

Using LEfSe analysis, we found two taxonomic nodes at M0 that can predict clinical response at M3 in our population: Betaproteobacteria class and, belonging to it, Burkholderiales order (Fig. 4A). AUC was 0.86 when testing Burkholderiales order abundance at M0 as a predictor of clinical response at M3 (Figure S2).

Similarly, at M3 many taxonomic levels appear to be differentially present when R and NR were compared (Supplementary Table 6). Again, after multiple test correction these results became not significant.

LEfSe analysis confirmed the results of the Wilcoxon test and indicated genus *Dialister* as the most strongly correlated with the responding patients at M3 in our population. Non-responders were characterized by the presence of genus *Salmonella* (Fig. 4B).

### Prediction of bacterial function

Additionally, we used PICRUSt^33^ to infer functions performed by microbiota using KEGG pathways database. This computational analysis allows to predict the metagenome functional content, matching genes of species found in our samples with a database that links gene sequences to metabolic functions. We found that at M0, microbiota of half of NR patients presented higher proportion of bacteria that have predicted genes involved in synthesis of lipopolysaccharides, ubiquinone and phenylpropanoids (Fig. 4C), that was not found in any R patients. Other non-responding patients have a profile of microbiota function very similar to that of responders. Patient 19 with an atypical phylum composition is the most distant compared to other patients also in terms of present microbial pathways.

## Discussion

### Strength and weakness of this study

Our study did not compare intestinal microbiota of patients with those of healthy people, so it does not establish the nature of the dysbiosis that is highly suspected to play a key role in the pathogenesis of SpA. Furthermore, we did not compare groups of patients treated either by anti-TNF-α or by all treatment but anti-TNF-α, so we cannot affirm that modifications of microbiota are specifically related to this pharmacological class. Moreover, although the majority of patients were treated by etanercept, the anti-TNF-α treatment was heterogeneous in our cohort making each conclusion more difficult, especially considering that *in vitro* and *in vivo* effects are different depending on molecules^35^. In particular, as we will discuss further, etanercept is expected to exert specific effects on microbiota. The small number of patients treated by other molecules than etanercept enabled us to focus on specific effects of this molecule. Some of our patients received other DMARDs than biologics; that could bias microbiota composition even if DMARDs consequences on microbiota composition are largely unknown. Generally, as our cohort was small, the power of our statistical analysis was limited.

We found that R patients had higher ASDAS scores and CRP at baseline; these results are coherent with previous studies^36,37^, which increases the external validity of our study.

We estimated intestinal microbiota by fecal microbiota analysis, even if the two are not equivalent^38^. However, using stool samples is the easiest way to assess intestinal microbiota, especially in patients without any digestive symptoms, and therefore do not require an endoscopy. Moreover, non-invasive methods are more suitable for developing biomarkers.

Our study is based on 16S rDNA sequencing, which allows only a partial view of microbiota focused on bacteria. Virobiota, mycobiota and eucaryota of intestinal tract are therefore not considered in this study, and dynamic interactions between all these microorganisms are consequently not encompassed, although many studies have shown implications of these actors in IBD physiopathology in particular and in human health in general^39^. Moreover, the quantification of taxonomic nodes is only relative since we use a ratio between the number of reads of a given taxonomic node and the total number of bacterial reads. In addition, 16S rDNA analysis is not discriminant enough for some close species. Quantitative PCR could allow more precise inter-sample comparisons. Although our predictive metabolic functional analysis is computational, it predicts the abundance of gene families with quantifiable uncertainty^33^.

We have not specifically screened inflammatory digestive manifestations in our population, so we have not evaluated the overlap between rheumatologic and digestive clinical manifestations, which could influence the results. We also did not take into account differing diets of patients.

### Impact of TNF-α inhibitors on microbiota

In this study we have not shown significant modification of particular taxa after treatment, probably due to low statistical power, but diversity seemed to be restored at M3 in NR.

Modifications of microbiota composition by TNF-α inhibitors could be caused either by indirect or direct effects. These treatments are well-known to heal and profoundly down-regulate inflammation in the wounded digestive mucosa, therefore restoring normal structure of digestive epithelium^40^ and control and tolerance functions toward mucosal microbiota. Thus, they could indirectly change microbiota composition.

More precisely, etanercept exerts a specific action on the host, which could be explained by its structure. Etanercept is a recombinant TNF receptor-Fc fusion protein^41^. Etanercept inhibits not only TNF-α but also soluble TNF-ß, aka lymphotoxin-α, while infliximab and adalimumab are monoclonal antibodies that are exclusively directed against TNF-α. Soluble form of lymphotoxin-α controls IgA induction in the lamina propria, and through this process controls microbiota composition^42^. Thus, the effects of etanercept could modify intestinal microbiota composition in a different way from the others TNF-α inhibitors *via* reduced IgA levels.

Direct action on the intestinal microbiota, *via* an inter-reigns regulation (inter-kingdom interaction), is suggested by several studies^43,44^. The existence of membrane-bound bacterial receptor to TNF-α has been anciently suspected, especially on gram negative bacteria^45^; in this study the presence of TNF-α seemed to increase virulence of *Shigella flexneri*. *In vitro* studies are needed to test the effects of TNF-α inhibitors used in clinical practice on bacterial gut commensals.

### Microbiota profiles as biomarkers of response to treatment

One of our more interesting results was that microbiota composition could predict clinical response to anti-TNF. Interestingly, such predictive capacity has been emphasized in multiple studies concerning various diseases. Overall diversity as well as presence or absence of specific taxa have been shown to be biomarkers of disease or response to treatment in other disorders. For example, intestinal microbiota has been proposed as a prognosis factor in colorectal cancer^46^. In advanced stages of colorectal cancer an increased colonic colonization by cyclomodulin-producing *E. coli* and enterotoxigenic *Bacteroides fragilis* and *Fusobacterium nucleatum* has been reported, suggesting a potential use of microbiota as a colorectal cancer prognosis biomarker^46^. Moreover, it has been suggested that chemotherapy toxicity could be dependent on microbiota composition^47^. Indeed, microbial-produced β-glucuronidases modulate irinotecan digestive toxicity^48^. In case of digestive tumors an intact gut microbiota is needed to obtain an optimal response to oxaliplatin. Indeed, microbiota together with the immune system augment intra-tumor oxaliplatin damages, modulating tumor oxidative microenvironment^49^. In melanoma, microbiota composition can predict resistance to immunotherapy-induced colitis and peculiar microbiota species can condition or potentialize CTLA4 or PD-L1 therapy effects^50–53^. Another recent study in ulcerative colitis patients treated by anti-TNF therapy revealed lower dysbiosis indices and higher abundance of *Faecalibacterium prausnitzii* in responders compared with non-responders at baseline. Furthermore, the authors showed that responders and non responders exhibited distinct mucosal antimicrobial peptides expression patterns^54^. Moreover, a recent study has shown that low concentrations of *F. prausnitzii* are correlated with early recurrence of Crohn’s disease after anti-TNF-α treatment interruption^55^. Studies in the field of rheumatology have shown that rheumatoid arthritis patients have altered gut and mouth microbiomes that are partly normalized after DMARDs treatment^56^ and could predict response to treatment^57^. However, the effect on the gut microbiome was shown to be moderate compared to the oral microbiome. Patients that responded well to treatment were characterized by a greater number of virulence factors before treatment and also by the reduction in *Holdemania filiformis* and *Bacteroides* sp. after treatment^56^. It should be noted that all these results concern other diseases than SpA. Given the differences in physiopathology, changes in the microbiota composition may also be different.

### Comparison with previous results

Similarly to Zhang *et al*.^56^, whose approach in rheumatoid arthritis patients did not find large differences in gut microbiota composition before and after non-biologic DMARDs treatment, in our small cohort we only observed moderate changes with limited statistical significance. However, those differences are consistent with the previous studies of microbiota in spondyloarthritis. First, we observed an increase of Proteobacteria in patient 19. This phylum has a low abundance in gut flora of healthy subjects and its increase have been associated with gastric bypass, metabolic disorders, inflammation and cancer^58^, which is consistent with our observation of unresolved inflammation in this patient. Second, we observed higher proportions of pathogenic and potentially pathogenic species in NR patients, specifically *Klebsiella oxytoca* and *Salmonella* sp. It has been shown before that AS patients produce higher levels of anti-*Klebsiella* and anti-*Enterobacter* secretory IgA, which have been hypothesized to interact with self-antigen HLA B27 and promote disease progression^59^. It is also known that some gastrointestinal (*Salmonella* sp., *Shigella* sp., *Yersinia* sp. and *Campylobacter* sp.) and urethral infections (*Chlamydia* sp.) can trigger reactive arthritis and up to 20% of those cases will develop AS within 10–20 years^60,61^. Considering that NR patients are characterized by the presence of *Salmonella* sp., especially at M3 sampling time, we hypothesize that this might be a contributing factor in their SpA development. Third, previous reports indicated the changes in Bacteroides associated with the state of the disease. The direction of the changes varies depending on the cohort^13,15^. In our study we observed that all NR patients showed a change in Bacteroides order: five of them had an increase and two a decrease, whereas R patients were not affected. This result suggests that the proportion of this bacteria order varies significantly in rheumatoid conditions.

Interestingly, R patients at M3 also exhibit higher proportion of *Lactobacillus delbrueckii*, species known to carry out the fermentation of kefirs and which has been previously proposed as probiotic in treatment for IBD^62^.

Magnusson *et al*. showed that abundance of *F. prausnitzii* increased in ulcerative colitis patients during induction therapy by anti-TNF-α in R, and not in NR^54^. A recent study in Crohn’s disease patients has shown changes in the microbiota composition after TNF-α inhibitor (adalimumab) treatment, with recovery of phylogroups (Firmicutes, Bacteroides and Actinobacteria) and decrease of *E. coli* during treatment^63^. We didn’t found comparable results in our study, but our patient population and treatment molecules were different.

We observed microbiota composition instability over time in NR. Major shifts in microbiota composition have been associated with diseases such as IBD and neurodevelopmental disorders^64^, but never with spondyloarthritis.

### Perspectives

It is now clearly established that subclinical or even symptomatic gut inflammation is associated with SA; nevertheless relationships between cause and effect remain to be established^65^. In the same way, we have only observed an association between microbiota composition clusters and clinical response, without presuming causality. Nonetheless, these results suggest that a specific fecal microbiota signature could be predictive of a good clinical response to the anti-TNF-α treatment, for which no biomarkers currently exist. It could be particularly clinically relevant to have a reliable test before the initiation of this type of treatment, to first avoid a delay in symptom relief, and second to ease the financial burden on health services. Prospective studies are required to confirm these results.

The stability of microbiota composition is considered to be critical for human health in general, and results from a competitive equilibrium within microbiota’s diverse bacterial, fungal and viral components^64^. Microbiota stability in patients could be a good prognostic factor in itself. This hypothesis would require longitudinal long-term studies in order to be confirmed.

## Conclusions

In this study based on microbiota composition analysis before and three months after treatment with TNF-α inhibitors, no specific taxon was observed to be consistently modified by the treatment. Nevertheless, each of NR patients displayed drastic differences before and after treatment in microbiota composition at order level, whereas R patients displayed only few mild differences, suggesting a higher stability of microbiota composition in R patients. Alpha-diversity of non-responders was lower at M0 compared to two other groups (PR and R) and this difference was rectified after anti-TNF-α treatment. High proportion of Burkholderiales at M0 was associated with the clinical response at M3, as high proportion of *Dialister* sp. at M3. All these results suggest possible biomarkers for anti-TNF-α efficacy in SpA patients.

## Electronic supplementary material

Supplementary information

Dataset1
