# Supplementary material for: Microbiota Composition May Predict Anti-Tnf Alpha Response in Spondyloarthritis Patients: an Exploratory Study
Source: Sci Rep. 2018 Apr 3;8:5446. doi: 10.1038/s41598-018-23571-4 (PMC5882885; doi:10.1038/s41598-018-23571-4)
Supplement: Supplementary file 1 — Supplementary information [file 41598_2018_23571_MOESM1_ESM.pdf]

MICROBIOTA COMPOSITION MAY PREDICT ANTI-TNF ALPHA RESPONSE IN SPONDYLOARTHRITIS PATIENTS: AN EXPLORATORY STUDY

Thomas Bazin[1,2] (thomas.bazin@u-bordeaux.fr), Katarzyna B. Hooks[3,4] (katarzyna.hooks@u-bordeaux.fr), Thomas Barnetche[5] (thomas.barnetche@chu-bordeaux.fr), Marie-Elise Truchetet[5] (me.truchetet@chu-bordeaux.fr), Raphaël Enaud[6,7,8] (raphael.enaud@u-bordeaux.fr), Christophe Richez[5] (christophe.richez@chu-bordeaux.fr), Maxime Dougados[9] (maxime.dougados@aphp.fr), Christophe Hubert[10,11] (christophe.hubert@u-bordeaux.fr), Aurélien Barré[3] (aurelien.barre@u-bordeaux.fr), Macha Nikolski[3,12] (macha.nikolski@u-bordeaux.fr), Thierry Schaeverbeke[1,5] (thierry.schaeverbeke@chu-bordeaux.fr)

SUPPLEMENTAL MATERIAL (separated file, online only)

Supplementary Table 1 - Clinical information for 19 patients recruited in the study.

Supplementary Table 2 - Summary of the sequencing, pre-processing and filtering of the 16S reads.

Supplementary Table 3 - Full normalized occurrence table based on taxonomic assignment by Tango

Supplementary Table 4 - Taxa significantly changed between samples at M0 and M3

Supplementary Table 5 - Significant differentially present taxa between R and NR samples at M0

Supplementary Table 6 - Significantly differentially present taxa between R and NR samples at M3

Supplementary Table 7 - Raw z-scores between M0 and M3 for all patients at Order level.

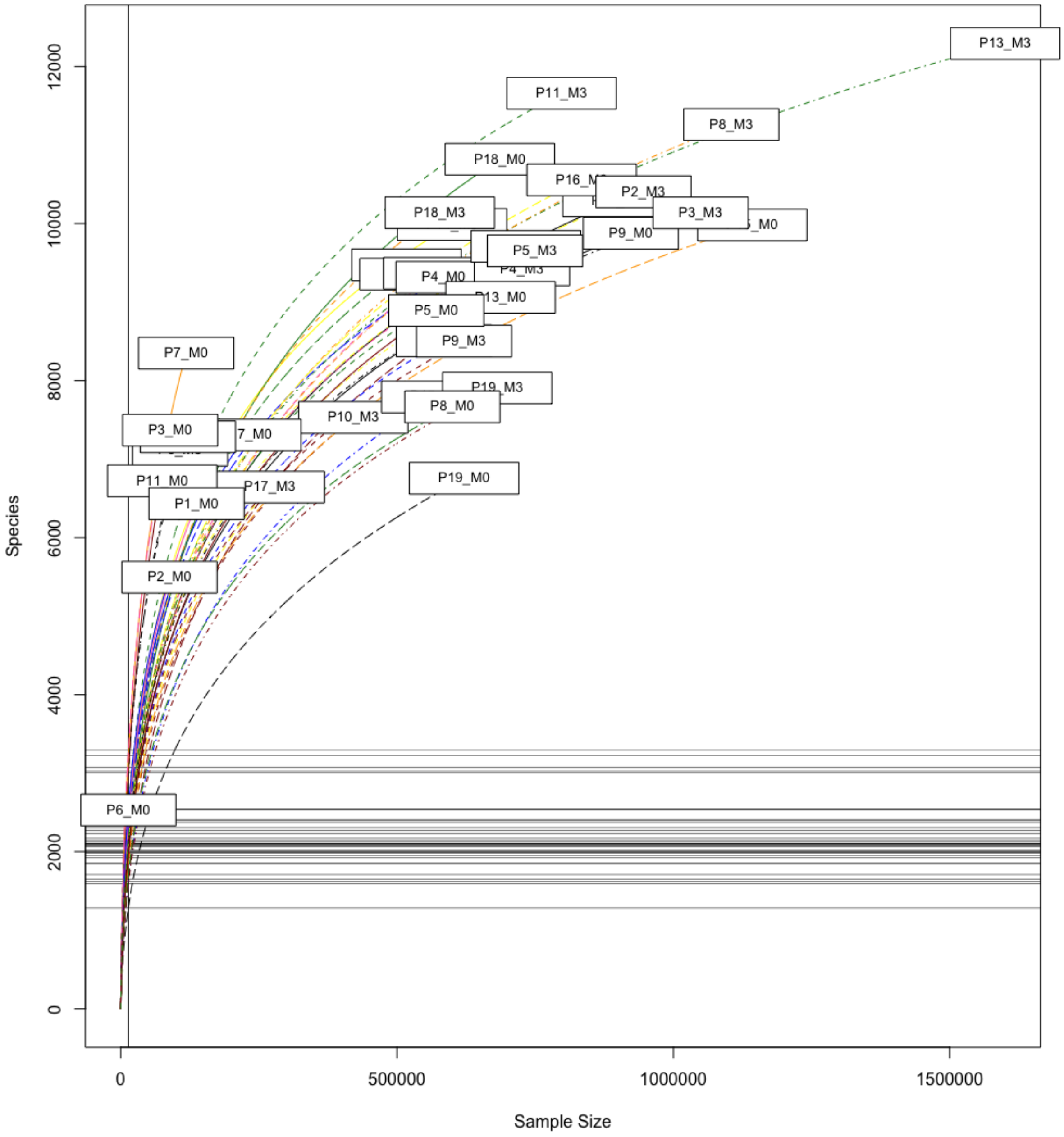

Figure S1: Rarefaction curves for all samples. Rarefaction curves based on GreenGenes OTUs were generated by vegan R package.

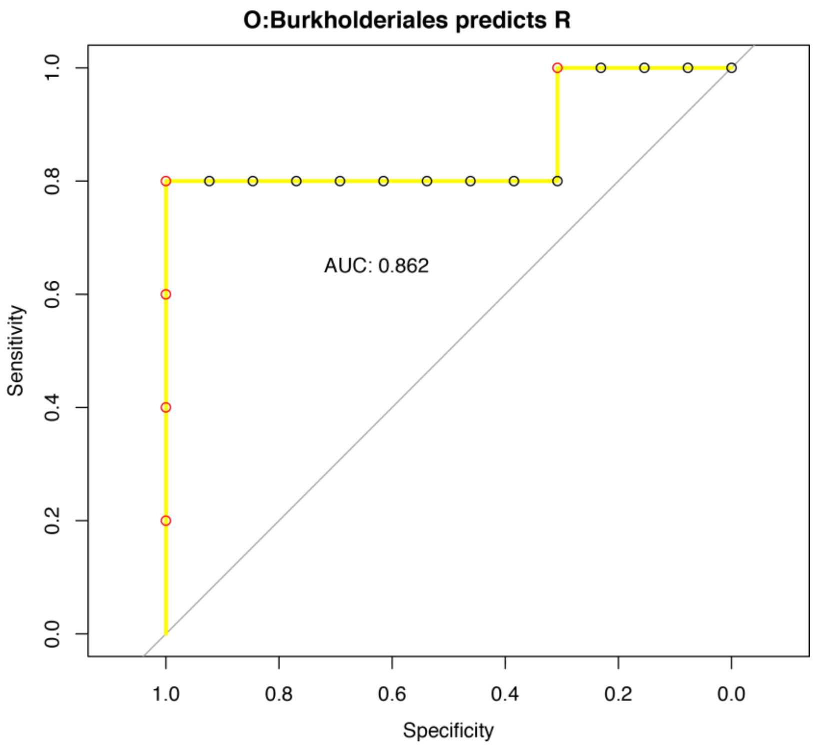

Figure S2: ROC curve of Burkholderiales order at M0 as a predictor of clinical response at M3. The curve was constructed by pROC package in R using the proportion of reads assigned by Tango to order Burkholderiales at M0 and clinical response measured at M3.
